# Thinking fast or slow? Functional magnetic resonance imaging reveals stronger connectivity when experienced neurologists diagnose ambiguous cases

**DOI:** 10.1093/braincomms/fcaa023

**Published:** 2020-03-02

**Authors:** Berry van den Berg, Anique B H de Bruin, Jan-Bernard C Marsman, Monicque M Lorist, Henk G Schmidt, André Aleman, Jos W Snoek

**Affiliations:** f1 Department of Experimental Psychology, University of Groningen, 9712 TS Groningen, The Netherlands; f2 School of Health Professions Education, Faculty of Health, Medicine and Life Sciences, Maastricht University, 6200 MD Maastricht, The Netherlands; f3 Department of Biomedical Sciences of Cells and Systems, University of Groningen, University Medical Center Groningen, Cognitive Neuroscience Center, 9700 AD Groningen, The Netherlands; f4 Department of Psychology, Faculty of Social Sciences, Erasmus University Rotterdam, 3000 DR Rotterdam, The Netherlands; f5 Center for Education Development and Research in Health Professions, University of Groningen and University Medical Center Groningen, P.O. Box 196, 9700 AD Groningen, The Netherlands; f6 Department of Neurology, Martini Hospital Groningen, 9700 RM Groningen, The Netherlands

**Keywords:** reasoning, dual-process theory, medical decision-making, medical expertise, applied cognitive psychology

## Abstract

For ∼40 years, thinking about reasoning has been dominated by dual-process theories. This model, consisting of two distinct types of human reasoning, one fast and effortless and the other slow and deliberate, has also been applied to medical diagnosis. Medical experts are trained to diagnose patients based on their symptoms. When symptoms are prototypical for a certain diagnosis, practitioners may rely on fast, recognition-based reasoning. However, if they are confronted with ambiguous clinical information slower, analytical reasoning is required. To examine the neural underpinnings of these two hypothesized forms of reasoning, 16 highly experienced clinical neurologists were asked to diagnose two types of medical cases, straightforward and ambiguous cases, while functional magnetic resonance imaging was being recorded. Compared with reading control sentences, diagnosing cases resulted in increased activation in brain areas typically found to be active during reasoning such as the caudate nucleus and frontal and parietal cortical regions. In addition, we found vast increased activity in the cerebellum. Regarding the activation differences between the two types of reasoning, no pronounced differences were observed in terms of regional activation. Notable differences were observed, though, in functional connectivity: cases containing ambiguous information showed stronger connectivity between specific regions in the frontal, parietal and temporal cortex in addition to the cerebellum. Based on these results, we propose that the higher demands in terms of controlled cognitive processing during analytical medical reasoning may be subserved by stronger communication between key regions for detecting and resolving uncertainty.

## Teaser

Here, experienced neurologists diagnosed straightforward and ambiguous cases while brain activity was measured using functional magnetic resonance imaging. Results show involvement, and differential connectivity of brain regions associated with the detection of ambiguity, attention and information content processing. The findings are consistent with a dynamic schematic anticipation model of medical reasoning.

## Graphical Abstract

**Graphical Abstract:**
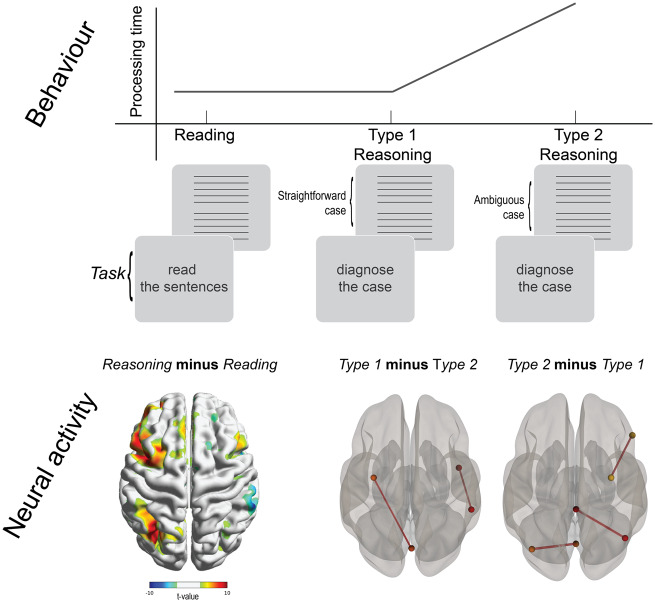

## Introduction

The cognitive processes and underlying neural mechanisms that shape reasoning are central to fast and accurate decision-making. Medical diagnosis is a particularly high stakes domain in which the accuracy of decision-making is imperative. During medical problem solving, experienced physicians are thought to rely primarily on recognition-based reasoning, using the so-called ‘illness scripts’ when diagnosing patients (Schmidt *et al.*, 1990; Schmidt and Rikers, 2007). These ‘illness scripts or schemas’ involve the retrieval of diagnostic hypotheses upon encountering typical symptoms of common diseases. For common clinical cases, medical experts’ reasoning is thought to be rapid, automated and heuristic (Type 1 processing). However, for less common cases, expert physicians are able to override this default manner of processing and revert to analytical processes (Type 2 processing) when encountering complex, ambiguous or contradictory symptoms (Pelaccia *et al.*, 2011). In these situations, medical reasoning is more elaborate as indicated by longer case response times, and better memory for clinical case information (Mamede *et al.*, 2010). Numerous theories based on research in social cognition, reasoning, judgement and decision-making exist that make a clear distinction between fast, automatic and intuitive reasoning (Type 1) and slow, effortful and analytical reasoning (Type 2) (Evans, 2003, 2008; Evans and Stanovich, 2013), the dual-process paradigm.

This distinction between these types of reasoning has been a useful concept in behavioural psychology and medical reasoning, but there is as of yet little empirical insight into their neural underpinnings. To shed more light on this, we conducted a functional MRI (fMRI) study on the neural correlates of Type 1 and Type 2 reasoning in medical decision-making.

Diagnostic reasoning by both experts and novices (medical students, junior doctors) is characterized by the same processes of hypothesis generation and verification (Brush *et al.*, 2017). Early hypothesis generation is based on pattern recognition, and the quality of this early hypothesis (or limited number of hypotheses) depends on knowledge gained through experience, which is highly personal. Expertise depends on the ability to rapidly access this highly personal body of knowledge, acquired in many years of clinical practice. In unfamiliar, atypical or otherwise complex cases, pattern recognition is insufficient and experts will have to turn to analytical forms of reasoning, using pathophysiological or conceptual knowledge. Research on clinical reasoning has revealed that physicians can be triggered into this more reflective mode of reasoning (versus an automatic reasoning mode) when encountering uncertainty when diagnosing clinical cases. This reflective reasoning is exemplified by longer processing times, but not reduced diagnostic accuracy, as the increase in processing time warrants optimal diagnostic reasoning (Norman *et al.*, 2017).

Here, this study thus addresses the question: is it possible to describe differences in neural correlates of different diagnostic strategies employed by experts in a circumscript field of expertise? Unlike in other studies in this field (Durning *et al.*, 2015; Hruska *et al.*, 2016), we were not interested in possible differences between neural correlates of reasoning strategies between medical experts and novices, or in novices (medical students) (Chang *et al.*, 2016), instead we focused on different reasoning strategies of medical experts themselves.

Medical reasoning is also a prototypical and relevant domain to empirically investigate the neural correlates of reasoning in a real-life setting. First, it is a high stakes domain: wrong decisions in a medical setting can have catastrophic consequences and important decisions often have to be made under time pressure. Moreover, task environments (e.g. evolution of symptoms and signs in a deteriorating patient) are dynamic and timely decisions must be made under the conditions of limited information, which may result in uncertainty about diagnosis and treatment.

On a neural level, dual processing in social cognition has been suggested to involve two distinct neural circuits: the reflexive system composed of the amygdala, basal ganglia and lateral temporal cortex and the controlled system involving the anterior cingulate cortex, the prefrontal cortex and the medial temporal lobe (Lieberman, 2000).

An alternative theory suggests that these two types of reasoning are actually executions of similar rules at different levels of automation, inferring activation of the same brain circuits and areas, even though the first cases are diagnosed faster than the latter (Kruglanski and Gigerenzer, 2011). The latter view is more in line with the current body of literature on the cognitive and neural underpinnings of brain functioning. The brain constantly makes predictions about future situations with as function to minimize the amount of information to be processed, an activity which is called predictive encoding (Feldman and Friston, 2010). In the case that unexpected information is encountered, a prediction error occurs. In terms of medical reasoning, this could occur when case information is ambiguous. For instance, a patient might present one or more symptoms that are more or less prototypical for a particular diagnosis. This information is processed, and the medical examiner comes up with a provisional, tentative diagnosis—the emergence of an illness script. These illness scripts can be described as the narrative of the daily manifestation of the underlying disease (Schmidt and Rikers, 2007). Incoming information from the patient is then matched to the illness script. Put differently, when the medical expert is given information about a case, the expert sets up an internal model of what other information can be expected and subsequent information (either directly present or searched for) is evaluated whether it strengthens (or even confirms) or weakens (even rejects) this model.

**Figure 1:**
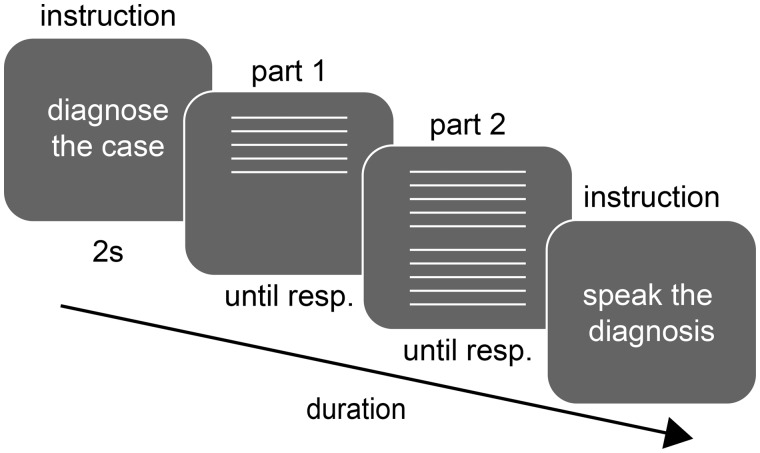
**Trial sequence for the medical cases**.

To study these processes in the human brain, we collected blood oxygen level-dependent imaging fMRI while 16 medical experts (licenced neurologists, >10 years of experience) processed and diagnosed both straightforward and ambiguous clinical cases (Fig. 1). This is in line with the daily practice of diagnosing patients by clinicians. Many patients in the setting of an outpatient clinic are ‘straightforward cases’: they present symptoms and signs more or less consistent with a well-known clinical pattern. Difficulties arise when symptoms or signs are atypical or absent when expected. In the dual-process paradigm, straightforward cases induce ‘automatic’ retrieval of a diagnostic hypothesis (i.e. Type 1 processing), while ambiguous cases require deliberate, reflective processing (i.e. Type 2 processing). For this study, carefully designed neurology cases were presented to the participating neurologists in two distinct versions, in both versions ultimately leading to the same final diagnosis. Clinical cases were 10 sentences long (*M*_words_ = 69; range= 60–82) and led to a single correct diagnosis. Type 2 cases were constructed by removing one to three sentences of Type 1 cases and replacing them with distracting patient information (noise), irrelevant to or conflicting with an early, provisional diagnosis, all information together ultimately leading to the same final diagnosis. The number of words for each Type 1 and corresponding Type 2 case was equal. The distorting information, intended to induce a shift from Type 1 reasoning into Type 2 reasoning, always followed three to four lines with identical information in the two conditions, which enabled participants to make an early hypothesis, a preliminary, tentative diagnosis. Although one cannot be sure that this manipulation results in an actual shift from Type 1 reasoning into Type 2 reasoning, it is reasonable to suppose that this occurs if accompanied by an increase in reaction times.

Participants in the fMRI study read and diagnosed 21 clinical cases (10 or 11 Type 1 cases, and 10 or 11 Type 2 cases) and read a baseline text to separate brain activation related to reading. After finishing each case, they vocalized the diagnosis in the scanner. Afterwards, participants provided confidence and difficulty ratings outside the scanner.

## Materials and Methods

The study had two phases. The first phase was a pilot study to validate the two different case types. The pilot study resulted in 21 cases that were used in the fMRI experiment during the second phase.

### Case validation pilot study

#### Participants

Seventeen native Dutch neurologists (recruited from the non-academic hospitals neighbouring Rotterdam) participated in the case validation pilot study (one woman, *M*_age_ = 53 years, range = 46–65 years). All participants had at least 10 years of working experience as a licenced neurologist (*M*_years_ = 20, range = 11–31 years) and practiced the full field of clinical neurology full time in a non-academic hospital. This latter requirement was a deliberate choice, as most neurologists in academic hospitals work as super specialists in a limited field, whereas in this study we focused on reasoning processes of experienced general neurologists, with broad expertise covering the entire field of clinical neurology. In the Netherlands, specialist training in neurology consists of 6-year undergraduate medical training followed by a 6-year residency phase. All neurologists volunteered to participate in this study without remuneration (other than a bottle of wine or a museum ticket).

### Stimuli, task, experimental design

The task of the participants was to diagnose 26 neurological cases presented on a laptop in E-prime (version 2.0). These clinical cases were constructed by two experienced neurologists, and each case had both Type 1 and Type 2 versions. Cases were constructed following defined requirements (broad coverage of the field of neurology; natural sequence of clinical information; cases had to be solvable with the information provided; approximately equal number of words). Each case consisted of 10 short sentences (mean number of words = 69; range = 60–82 words). Type 1 cases were more or less prototypical, where most items were exemplary for and a least consistent with the correct final diagnosis. Type 2 cases were adapted by replacing one to three sentences with ambiguous patient information (noise) to elicit slow, analytical reasoning. In the Type 2 cases, a similar tentative diagnosis was possible within the first three to four items without the need of overt reasoning. To provoke analytical reasoning, usually the fourth and/or fifth sentences contained noise information; sometimes an additional noise item was added in the latter part of the text. Depending on the cases, care was taken to assure that, although atypical, the Type 2 cases were solvable using the information provided (for two case examples and a list of case diagnoses, see the Supplementary material).

Following a practice case, participants diagnosed, self-paced, 26 cases, ensuring that there was a balanced distribution of Type 1 (13) and Type 2 (13) cases. Each case can be considered as an individual trial and consisted of the presentation of five lines of white text on a black background at the upper half of the screen. After participants had read the five lines of text, they pressed a button and the remaining five sentences were provided below the first five lines. When participants reached a diagnosis, they pressed a button to type this diagnosis in the next screen. They also indicated (on a scale from 0 to 100) how confident they were with their diagnosis, how much effort it took them and how difficult the case was.

Diagnostic accuracy (0 = incorrect diagnosis, 1 = partially correct diagnosis, 2 = correct diagnosis), case processing time (s) and subjective measures of confidence, effort and complexity were analysed across participants to allow for case validation. Diagnostic accuracy was determined by J.W.S. and an independent fellow neurologist. The Spearman correlation between the raters’ scores was 0.82. Through discussion, agreement was reached on all rating disagreements. A unified rating of diagnostic accuracy was obtained that was also used in the final fMRI experiment. The following case selection criteria were applied to determine cases for the fMRI experiment: (i) mean diagnostic accuracy >1 and (ii) processing time Type 2 >  Type 1. Finally, confidence, effort and difficulty were analysed to further validate the distinction between Type 1 and Type 2 cases and check that effort and difficulty were higher for Type 2 cases than for Type 1 cases and confidence was lower for Type 2 cases than for Type 1 cases. These criteria led to the removal of five cases, leading to a total of 21 cases to be used in the fMRI experiment. For these 21 cases, processing times during the pilot study were on average 41% longer in the Type 2 version than in the Type 1 version (see Table 1).

**Table 1:** Mean (SD) case processing times, diagnostic accuracy, confidence, difficulty and effort ratings for the case validation pilot study and the final fMRI study separated for Type 1 and Type 2 cases

	Case pilot validation study			fMRI study		
	Type 1 cases, mean (SD)	Type 2 cases, mean (SD)	Type 2 − Type 1, *t*-value (df = 16)	Type 1 cases, mean (SD)	Type 2 cases, mean (SD)	Type 2 − Type 1, *t*-value (df = 15)
Case processing time (s)	39 (14)	55 (17)	2.88^a^	21.6 (6.2)	26.3 (9.0)	4.74^b^
Diagnostic accuracy	1.87 (0.12)	1.64 (0.12)	−5.05^b^	1.81 (0.16)	1.61 (0.26)	−2.72^a^
Confidence	80 (7)	69 (9)	−4.70^b^	81 (11)	70 (14)	−2.81^a^
Difficulty	32 (15)	46 (15)	2.30^a^	26 (12)	40 (15)	4.74^b^
Effort	32 (13)	45 (14)	2.51^a^	NA^c^	NA^c^	NA^c^

Notes: The data for the case pilot validation study include five cases that were not selected for the final fMRI study. Diagnostic accuracy was scored as 0 (incorrect diagnosis), 1 (partially correct diagnosis) or 2 (correct diagnosis). Confidence and difficulty were provided on a Likert-type scale from 1 (not at all confident/difficult) to 7 (very confident/difficult) in the fMRI experiment and transformed to a percentage for this table.

a Difference between Type 1 and Type 2 cases is significant at *P* < 0.05.

b Difference between Type 1 and Type 2 cases is significant at *P* < 0.001.

^c^NA: not available.

### fMRI experiment

#### Participants

Seventeen native Dutch neurologists were recruited for the fMRI experiment. Participants were recruited by an advertisement on a national neurological conference and via email lists. One participant was excluded because he had a metal implant of unknown material after a fractured chin, which made MRI scanning impossible. Therefore, 16 neurologists participated in the final experiment (four women; *M*_age_ = 51 years, range 46–57 years). All participants had at least 10 years of working experience as a licenced neurologist (*M*_age_ = 18 years, range = 10–28 years) and practiced general neurology on a full-time basis in a non-academic hospital. The study was conducted in according to protocols approved by the Medical Ethical Committee of the University Medical Center Groningen. Prior to the start of the experiment, participants gave informed consent in accordance with the Declaration of Helsinki.

### Stimuli, task and experimental design

Each participant underwent an fMRI scan where two runs were recorded, intermitted by an anatomical recording. Participants were asked to diagnose 21 neurological cases (*M*_word__count_= 69, range = 60–82), selected through the case validation pilot study. Participants each processed and verbally diagnosed 10 or 11 Type 1 cases, and 10 or 11 Type 2 cases (see Fig. 1 for an overview of the trial sequence for a case to be diagnosed). Case distribution was counterbalanced across participants. Their diagnosis was transmitted by means of an in-bore microphone and recorded using a digital dictaphone after each case.

The experimental procedure was the same as the pilot study, except that participants also read cloze probability sentences belonging to a control condition (10 trials consisting of 10 sentences each with seven or eight words), which were randomly interspersed between cases. Cloze probability sentences are lexically and semantically correct passive sentences (but the sentences did not form a coherent story together). They were used as a reading control condition as these sentences require minimal cognitive processes (Kutas and Hillyard, 1984; Delong *et al.*, 2011). Finally, a screen showing only a red fixation cross was presented 10 times, randomly intermixed between the cases and reading trials, as a baseline condition (duration 15–25 s, timing randomly sampled from a uniform distribution).

All stimuli were programmed and presented using E-prime (version 2.0) and displayed using a Barco liquid crystal display projector G300 (Barco, Kortrijk, Belgium) on a translucent display at a resolution of 1024 × 768 pixels. The dimensions of the translucent display were 44 cm × 34 cm. This subtends a visual angle of 32° × 25.5° for the entire screen. Via a 45° tilted mirror, placed on the top of the head coil, the participant was able to see the entire presentation display. The distance from the eyes to the screen (via the mirror) was 75 cm. After the fMRI experiment was completed, the experimenter brought the participant to another room and asked them to rate the various cases using a 7-point scale on both difficulty and confidence.

### fMRI data acquisition

Scanning was performed using a 3.0-T MRI Scanner (Philips, Best, The Netherlands) with a 32-channel SENSE head coil. Functional recordings (echo planar images, axial slices recorded in a descending manner) were made using the following parameter settings: flip angle: 70°; echo time: 30 ms; repetition time: 2000 ms, field of view: 224 mm × 136.5 mm × 224 mm; 39 slices were acquired in descending order (slice thickness of 3.5 mm, in-plane resolution of 3.5 mm). Two sessions were recorded with variable number of volumes since the task was semi-self-paced (all sessions were completed within 15 min). Between the two sessions, a high-resolution anatomical scan was recorded. The anatomical T1 was made with an in-plane resolution of 1 mm × 1 mm, contained 160 slices and recorded transversal. Field of view was 232 mm × 170 mm × 256 mm.

### fMRI data analysis

All analyses were performed in MATLAB (2015) (MathWorks, Natick, MA, USA). Magnetic resonance images were analysed using SPM12 (http://www.fil.ion.ucl.ac.uk/spm/). Preprocessing consisted of slice time correction, realignment to correct for participants movement, co-registration to align all functional data to the participant’s anatomical T1, normalization to map all images to Montreal Neurological Institute space and spatial smoothing with a Gaussian kernel of 8 mm (full width at half maximum). The images for each functional session were high-pass filtered at 0.008 Hz. Onsets of both case types and cloze probability sentence were consequently used as regressors for the first-level analysis together with the processing time (i.e. the moment the participant pressed the button to continue or give the diagnosis). We did not exclude cases from the fMRI analysis based on their accuracy, which could also be partly accurate.

Additional preprocessing steps for the connectivity analysis including denoising regressors in the design that correspond to white matter, CSF and mean global signal to remove related variance, these signals were derived from the subject’s anatomical image using segmentation in SPM12. In the CONN toolbox region of interest (ROI)-to-ROI analyses, time courses of each voxel in each ROI are averaged to create an ROI-level time course and then each ROI time course is correlated Fisher’s transformed bivariate coefficient representing the functional connectivity between each region. The CONN toolbox extracts five temporal components from the segmented CSF and white matter, which were entered as confound regressors in the task generalized linear model inside CONN (following the ‘default’ strategy implemented in the CONN toolbox and followed by others) (Damme *et al.*, 2019; Deverdun *et al.*, 2019).

### Statistical analysis

At the participant level of the fMRI analysis, contrasts were calculated for Type 1 cases versus baseline, Type 2 cases versus baseline and reasoning (Type 1 and Type 2 cases together) versus reading. At the second level of the fMRI analysis, a *t*-test of each of these contrasts were performed and reported at *P* < 0.05, corrected for family-wise error rate (FWER). Figures were made using BrainNet viewer (Xia *et al.*, 2013). To give a broader overview of potentially relevant brain areas, we also included differences in activation between Type 1 and Type 2 cases that reach a statistical threshold of *P* < 0.005 at the uncorrected level.

Finally, functional connectivity analysis was performed based on parcellated brain regions using the CONN toolbox (version 18.b—https://web.conn-toolbox.org). The CONN toolbox is based on the CompCor method and performs an ROI-to-ROI condition-dependent correlation of the timeseries derived from the residuals of the blood oxygen level-dependent imaging timeseries (Fair *et al.*, 2007; Whitfield-Gabrieli and Nieto-Castanon, 2012). The ROI parcellation in Conn is based on the Harvard–Oxford atlas areas and resulted in 132 predefined ROIs. A second-level test on functional connectivity between parcellated brain regions was performed for the contrast of Type 2 versus Type 1 cases. Connections were reported using *P* < 0.05, corrected for false discovery rate.

For both the case validation pilot study and the fMRI experiment, the mean case processing time, diagnostic accuracy and the various ratings [confidence, difficulty and effort (only for the case validation pilot study)] were compared between Type 1 cases and Type 2 cases using a paired two sample *t*-test and considered statistically significant for *P* < 0.05.

### Data availability

The behavioural data from the pilot study and fMRI study are available through the first author (berry.van.den.berg@rug.nl). The fMRI data are available through the University Medical Center (j.b.c.marsman@umcg.nl).

## Results

### Behavioural results

**Figure 2:**
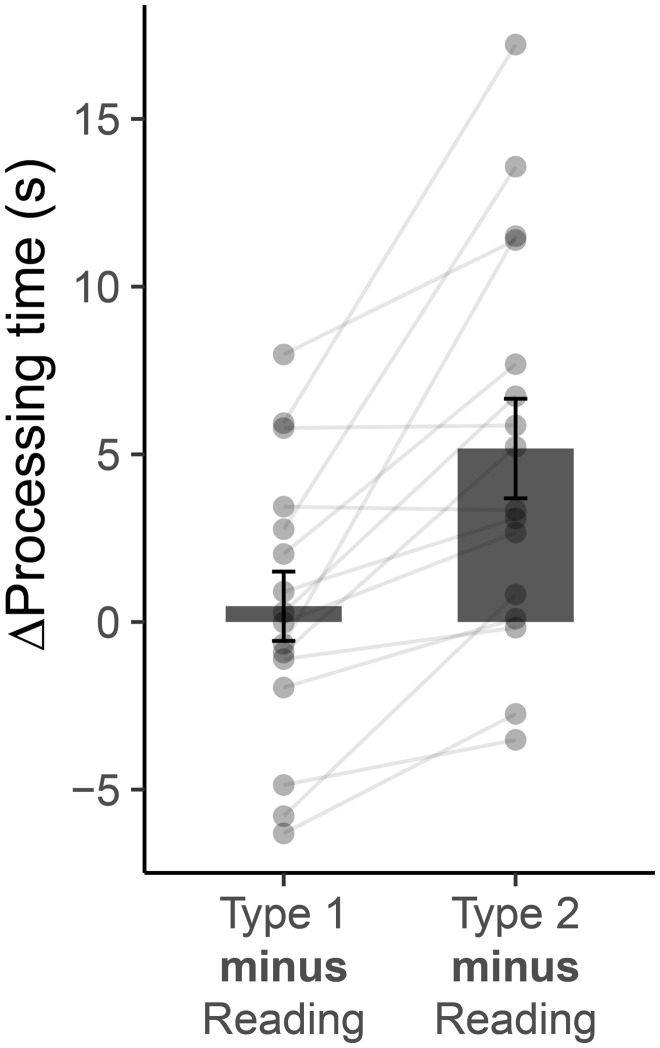
**Behavioural results.** Bar graphs represent the difference between the response time of diagnosing cases (Type 1 and Type 2) and reading cloze probability sentences. Error bar reflects mean ± SEM.

Significant differences in case processing time (mean reaction time_Type 1_ < mean reaction time_Type 2_, see Table 1 and Fig. 2), diagnostic accuracy and subjective difficulty ratings between the Type 1 and Type 2 cases were found, indicating typical behavioural differences between both forms of reasoning. Case processing times were on average 18% shorter for the Type 1 version than for the Type 2 version. Importantly, the processing time of the Type 1 cases was not significantly different to the reading time of the reading of cloze probability sentences [*t*(15) = 0.45, *P* = n.s.], but Type 2 cases were processed slower compared with reading [*t*(15) = 3.48, *P* = 0.0033]. Even though experts took less time to process clinical cases while in the scanner compared with the pilot group outside the scanner, their performance in diagnostic accuracy and ratings on difficulty or confidence were similar in both Type 1 and Type 2 cases.

### fMRI results

**Figure 3:**
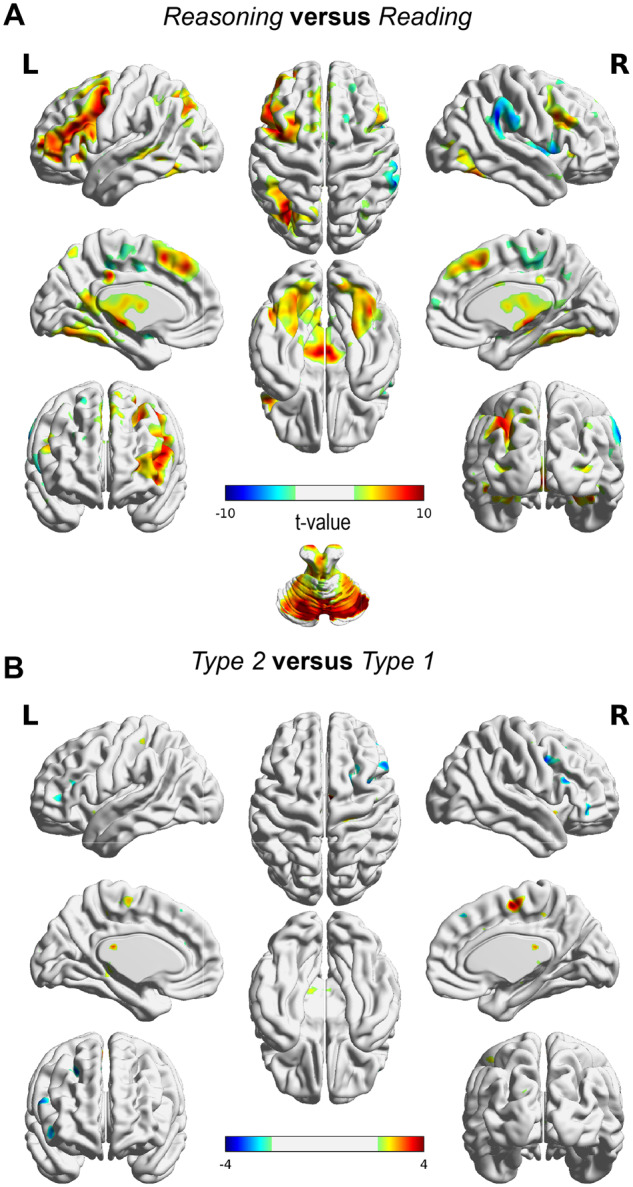
**Blood oxygen level-dependent imaging activity differences.** (**A**) Reasoning (Type 1 cases and Type 2 cases together) showed activity in caudate nucleus, left prefrontal, parietal and cerebellar regions. (**B**) Activity difference map between Type 1 and Type 2 cases. No significant difference was found at *P* < 0.05 (FWE corrected).

Our first analysis focused on the difference between reading cloze probability sentences and diagnosing cases to check whether the reasoning conditions (Type 1 and Type 2 cases combined) resulted in the activation of brain areas generally associated with reasoning (Table 2 and Fig. 3). Notably, this contrast revealed several brain areas with increases in blood oxygen level-dependent imaging signal. In particular, this pertained activation of the left frontal and left parietal regions, caudate nucleus, superior frontal gyrus and cerebellum.

**Table 2:** Brain regions where significant differential activity was found between reasoning (both Type 1 and Type 2 cases) and reading

Contrast	Region	Peak voxel coordinates (*x*, *y*, *z* in MNI space)			*t*-Value	Cluster size (*n* voxels)
Reasoning > reading	Cerebellum	−4	−78	−32	16.94	5487
	Left inf. and sup. frontal	−48	6	30	13.98	1000
	Left thalamus	−8	−8	4	11.72	136
	Brainstem	4	−34	−22	11.71	425
	Right caudate	16	−6	22	10.82	208
	Left hippocampus	36	−24	−8	10.45	20
	Left parietal cortex	−34	−76	38	10.30	243
	Left frontal cortex	−10	38	42	10.10	48
	Right thalamus	14	−6	0	8.74	42
	Left insula	−30	22	−4	8.55	29
Reading > reasoning	Right parietal lobule	68	−42	30	13.20	137
	Right insula	389	−12	−2	8.41	13
	Right anterior insula	40	4	4	7.69	13

Only cluster size >10 and *P* < 0.05 (FWER corrected) are reported.

FWER: family-wise error rate; MNI: Montreal Neurological Institute.

Our next analysis focused on testing the hypothesis that differential areas would be involved when processing Type 1 versus Type 2 cases. We found no observable difference in the magnitude of the blood oxygen level-dependent imaging response between these two different types of cases. Lowering the significance threshold (*P* < 0.005 uncorrected) revealed several areas with trend-level differential activity between the different case types (Table 3). Notably, for Type 1 > Type 2, these areas were slightly anterior to left cingulate gyrus, supplementary motor area and slightly anterior to right cingulate gyrus. For Type 2 > Type 1, these areas included the right putamen, slightly inferior to right anterior cingulate, slightly inferior to left anterior cingulate and various regions in the (orbito) frontal cortex. Given the lack of significance at FWE-corrected level (*P* < 0.05) activity, differences should be interpreted with caution.

**Table 3:** Brain regions where differential activity (*P* < 0.005, not FWE corrected) was found between Type 1 and Type 2

Contrast	Region	Peak voxel coordinates (*x*, *y*, *z* in MNI space)			*t*-Value	Cluster size (*n* voxels)
Type 1 > Type 2	Anterior to left cingulate gyrus	20	−44	22	4.71	54
	Supp. motor area	8	−8	56	4.50	144
	Anterior to right cingulate gyrus	−4	−32	20	4.13	16
	Right primary motor cortex	18	−26	64	3.56	28
	Right temporal cortex	46	−34	−10	3.55	16
	Cerebellum	−16	−48	−28	3.48	13
	Right insula	40	10	−8	3.43	11
	Left parietal	30	−54	28	3.37	16
	Right dorsal posterior cingulate	−18	−52	36	3.26	10
Type 2 > Type 1	Right putamen	24	0	8	5.57	140
	Inferior to left anterior cingulate	12	22	18	4.48	47
	Inferior to right anterior cingulate	−8	16	22	3.60	26
	Left frontal cortex	38	2	40	3.58	34
	Left frontal cortex	22	14	50	3.50	74
	Right orbitofrontal cortex	−36	40	0	3.46	18
	Left frontal cortex	58	20	22	3.32	36
	Left orbitofrontal cortex	46	36	−10	3.28	15

Only cluster size >10 and *P* < 0.005 (uncorrected) are reported.

MNI: Montreal Neurological Institute.

**Figure 4:**
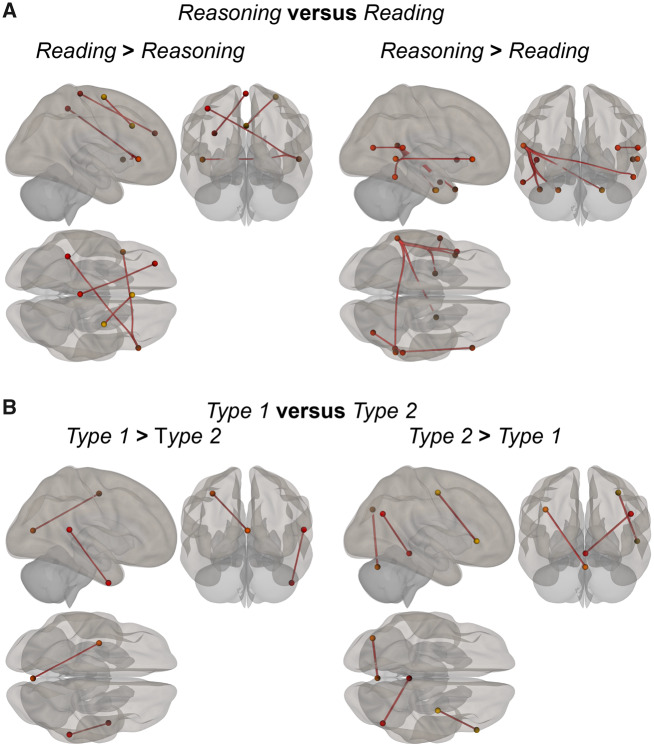
**Connectivity differences.** (**A**) Connections between regions which are significantly more involved reasoning compared with reading. (**B**) Connections between regions significantly involved in diagnosing the different types of cases.

Connectivity results for reasoning versus reading showed various connections that differed between both conditions. Most prominent locations for reasoning were regions residing in bilateral temporal gyrus and frontal gyrus. For reading, these included regions in the frontal and motor cortex (Fig. 4A).

Finally, we investigated connectivity differences in brain activity during the diagnoses of Type 1 and Type 2 cases (Fig. 4B). We found two connections stronger for Type 1 compared with Type 2: right posterior superior temporal gyrus with right anterior inferior temporal gyrus and left medial visual regions with left precentral gyrus (see also Table 4). For Type 2 cases, we found stronger connections between inferior temporal gyrus and middle temporal gyrus, superior temporal gyrus and frontoparietal regions, i.e. posterior parietal cortex, vermis in the cerebellum and middle temporal gyrus (Fig. 4 and Table 4).

**Table 4:** Connectivity results for reasoning versus reading and Type 1 versus Type 2

Region 1	Region 2	*t*-Value	*P*-uncorrected	*P* (FDR < 0.05)
Reasoning > reading				
Posterior superior temporal gyrus left	Anterior parahippocampal gyrus, left	5.25	<0.0001	0.008
Posterior superior temporal gyrus left	Anterior parahippocampal gyrus, right	3.67	0.0011	0.0312
Posterior superior temporal gyrus left	Anterior medial temporal gyrus	4.62	0.0002	0.0137
Posterior superior temporal gyrus left	Occipital right inferior temporal gyrus	4.34	0.0003	0.0159
Posterior superior temporal gyrus left	Temporal pole left	3.86	0.0008	0.0312
Inferior frontal gyrus	Occipital right medial temporal gyrus	4.76	0.0001	0.0206
Reading > reasoning				
Rostral prefrontal cortex	Precuneus	5.19	0.0001	0.009
Frontoparietal cortex	Posterior superior temporal gyrus	4.37	0.0003	0.0446
Inferior frontal gyrus	Parietal operculum	4.38	0.0003	0.0445
Type 1 > Type 2				
Posterior superior temporal gyrus	Anterior inferior temporal gyrus	4.41	0.0003	0.0416
Medial visual region	Precentral gyrus left	4.32	0.0003	0.0499
Type 2 > Type 1				
Inferior frontal gyrus right	Pre-central gyrus right	5.38	<0.0001	0.0063
Right lateral parietal	Vermis region 3	5.17	0.0001	0.0093
Left lateral parietal	Vermis region 7	5.06	0.0001	0.0116

FDR: false discovery rate.

## Discussion

The aim of the current study was to examine neural underpinnings of reasoning in expert medical reasoning. To do so, in this study, expert general neurologists (>10 years of experience) diagnosed patient cases while concurrently brain activity was being recorded (fMRI). We focused on the reasoning processes underlying two types of medical cases: one type that induced diagnostic uncertainty (Type 2 cases) and compared neural activation and functional connectivity during reasoning to those cases that were relatively straightforward (Type 1 cases). Our study is novel in that we presented cases that, in all likelihood, induced a shift in reasoning strategy without instructing the participants to do so. Although the highly artificial study situation inside an fMRI scanner of course differs extremely from the real clinical situation in an outpatient clinic, the mix of common and uncommon clinical cases is natural to the clinician, who sees many typical and untypical patients every week. In this respect, our study differs from studies on cerebral activation during the solving of clinical multiple-choice questions (Durning *et al.*, 2015; Chang *et al.*, 2016; Hruska *et al.*, 2016). Our study also differs from these studies in that our participants were all experts in their field, experienced neurologists, who served as their own controls.

The neural correlates of the reasoning of experienced neurologists were comparable to results from other studies in participants with other backgrounds. Specifically, during reasoning (versus reading cloze probability sentences), neurologists showed greater activity in the caudate, left frontal and parietal brain regions. These areas have been shown in previous studies to be active during reasoning and medical reasoning (versus control) (Rodriguez-Moreno and Hirsch, 2009; Chang *et al.*, 2016; Hruska *et al.*, 2016). Combined with our results, this suggests that the specific diagnostic processes and brain areas used by neurologists during medical reasoning resemble those found during other types of reasoning (e.g. deductive reasoning). Perhaps surprisingly, we found a large ensemble of cerebellar areas active during reasoning compared with reading. The cerebellum has long been known to be involved in motor control and is thought to serve a major role in working memory, cognitive control and executive functioning (Durisko and Fiez, 2010; Schmahmann, 2019).

Regarding differences between the reasoning conditions: on a behavioural level, participants responded slower and rated Type 2 cases (as compared with Type 1) as more difficult. Interestingly, Type 1 cases had similar processing time as compared with reading cloze probability sentences. From a conceptual level, the time spent on diagnosing cases consists of both reading the textual sentences and the diagnostic process. The observation that participants had slower processing time for Type 2 cases (versus both reading and Type 1 cases) suggests that the Type 2 cases did evoke additional thinking processes. On a neural level, we did not observe strong regional activation differences of brain areas. Thus, there may be considerable overlap in neural substrates subserving any kind of diagnostic inference. Of note, we did find trend-level differences in the magnitude of brain activation, in particular in posterior (greater for Type 1) and frontal (greater for Type 2) regions. More specifically, the posterior involvement might be related to response preparation for straightforward cases and the frontal involvement might be related to the processing of uncertainty for ambiguous cases. Clearly, this needs further investigation, as these results are tentative given the low statistical power.

The reasoning task used here was specifically designed for a group of highly trained medical experts. On one hand, this allowed for a high degree of ecological validity regarding the reasoning processes evoked by the various cases. On the other hand, a limitation of the study is that the selection of this specialized group resulted in relatively low statistical power due to the limited number of participants and female participants that we could include. In addition, in the scanner, our group of experienced neurologists responded much faster compared with the neurologists in the case validation pilot study. However, the relative difference in processing time between Type 1 and Type 2 (∼20%) was similar.

Connectivity results between the two types of cases (Fig. 4B) did support the hypothesis of differential network involvement in diagnostic processing of Type 1 and Type 2 cases. More specifically, whereas Type 1 cases evoked stronger connectivity within the temporal lobe and between occipital and parietal areas, Type 2 cases evoked stronger connectivity between frontal, temporal and parietal areas in addition to a connection between posterior parietal cortex and the cerebellum. The temporal and parietal regions may be part of networks subserving memory and attention, respectively, which are relevant for both types of processing, albeit in different ways.

On the other hand, Type 2 cases clearly involved stronger connectivity for brain regions that have been identified as key nodes in brain networks for executive functioning. This may involve cognitive effort and conflict processing (Kutas and Federmeier, 2011; Kalisch and Gerlicher, 2014). Of note, the cerebellum has been shown to be involved in cognitive functioning (Schmahmann *et al.*, 2019), including executive functions such as cognitive control. Interestingly, a recent study found involvement of connectivity of cerebellum and parietal cortex during repeated behavioural information uptake informing about personality traits of different persons (Van Overwalle *et al.*, 2017), which could be considered to be similar to processing diagnostic information about patients.

On a conceptual level, during the collection of information about a clinical case, one or more hypotheses or predictions about the most likely—but as yet provisional—diagnosis are constructed early in the diagnostic process, based on the information that has been received thus far (Schmidt and Rikers, 2007). If the next encountered information is in conflict or does not support the current hypothesis, this conflict is detected and the initial hypothesis has to be revised and updated based on the new information. As such, we interpret the role of prefrontal areas as key in the detection of ambiguous information (Mohr *et al.*, 2015), which may extend to the process of generating a provisional diagnosis. The main difference in neural activity between Type 1 and Type 2 cases was found to concern increased connectivity between the frontal and parietal cortex, anterior and posterior temporal cortex and parietal–cerebellum connection. This connectivity finding fits well into a wider body of literature regarding the detection, processing and resolution of uncertainty (Yoshida and Ishii, 2006; Goñi *et al.*, 2011; FeldmanHall *et al.*, 2019). The lateral prefrontal cortex has been shown to be active in redecision processing (revisiting one’s initial decisions) and to be scaled with decision uncertainty reduction and correlated with individual accuracy changes in a rule-based decision-making task (Qiu *et al.*, 2018).

Based on the connectivity differences between Type 1 and Type 2 cases, we propose that the main difference in neural mechanisms between the reasoning strategies during solving these distinct types of cases is the regulation of information flow. We speculate that following the detection of ambiguity in Type 2 cases, the areas that are key in executive functioning (e.g. lateral prefrontal cortex) regulate attentional control and the types of (ambiguous) information that are important to attend to. We propose that subsequent differential communication between the frontal, parietal and temporal regions (important for memory retrieval of expert knowledge) that are involved in more fundamental cognitive mechanisms gives rise to the overarching differences in reasoning processes.

We propose a more integrative dynamic view of medical reasoning. Instead of grounding this view into categorically different types of reasoning (i.e. dual processes), a better description can be based on the (old, but not obsolete) information-processing psychology concept of ‘schematic anticipation’ as described by Otto Selz in the early 20th century (de Groot, 1965), and the more recent neuroscience predictive encoding theories. According to Selz, when setting a concrete goal (i.e. establishing a correct diagnosis), a ‘schematic anticipation’ of the consequence(s) of reaching this goal is always implied. This schematic anticipation of the solution is the starting point for further reasoning processes. The ease and speed of these reasoning processes are determined by the force (completeness) of the missing data necessary to close the gap between the anticipated and final solution.

Future research should investigate the possible underlying neural mechanisms in more detail. For example, a modern neuroscience counterpart of the concept of schematic anticipation is the neural predictive encoding theory (Friston, 2010, 2018; Clark, 2013), where a hypothesis is generated by the brain that may need to be adapted in the face of new information.

In summary, in this study, we found that the differences between processing straightforward (Type 1) and ambiguous (Type 2) cases were processing time, diagnostic accuracy and neural connectivity between areas that may subserve a higher degree of controlled cognitive processing (i.e. executive functions). The differences between Type 2 and Type 1 may not primarily lie in discrete activation levels of isolated brain regions, but rather in the communication or connectivity between key nodes in relevant networks. These may underlie the detection of uncertainty, generation of hypotheses and subsequent revisions of a provisional diagnosis. In clinical settings, this means that the clinician anticipates a final diagnosis by first creating an early provisional diagnosis. When subsequent case information is inconsistent or inconclusive with this provisional diagnosis, the brain engages in more elaborate controlled processing to compare and integrate externally presented information to knowledge from memory. Thus, in our opinion, a cornerstone of expert reasoning lies in enhanced neural communication, necessary to dynamically revise a provisional diagnosis during the collection of ambiguous, and possibly conflicting clinical information.

## Supplementary material

Supplementary material is available at *Brain Communications* online.

## Supplementary Material

fcaa023_Supplementary_Data

## Abbreviations

fMRI =: functional MRI
ROI =: region of interest
