# Supplementary material for: Thinking fast or slow? Functional magnetic resonance imaging reveals stronger connectivity when experienced neurologists diagnose ambiguous cases
Source: Brain Commun. 2020 Mar 2;2(1):fcaa023. doi: 10.1093/braincomms/fcaa023 (PMC7425520; doi:10.1093/braincomms/fcaa023)
Supplement: fcaa023_Supplementary_Data [file fcaa023_supplementary_data.pdf]

Supplementary Material

Herpes simplex encephalitis Type 1 version *(60 words in Dutch)*

1. female, 38 yrs old
2. admitted because of a generalized seizure
3. malaise for one week, irritable, headache
4. on admission meningeal irritation
5. body temperature 38,5 degrees Celsius *(=101,3 degrees Fahrenheit)*
6. she is aphasic and confused
7. MRI: swollen temporal lobe on the left side
8. CSF: 340x 10^9 cells, mostly mononuclear
9. EEG: irritative activity left frontotemporal region
10. positive HSE* PCR in CSF

Herpes simplex encephalitis Type 2 version *(61 words in Dutch)*

1. female, 38 yrs old
2. admitted because of a generalized seizure
3. malaise for one week, irritable, headache
4. past history: splenectomy
5. on admission meningeal irritation
6. body temperature 38,5 degrees Celsius
7. she is aphasic and confused
8. CT normal (too restless for MRI)
9. CSF: 340x 10^9 cells, mostly mononuclear
10. positive HSE PCR in CSF

Note that most items are identical, except items 4 and 8 in the Type 2 version, while item 9 is in the Type 1 version only. The previous splenectomy (item 4) is a noise-item, as it suggests the possibility of a bacterial and not a viral intracranial infection.

* HSE: herpes simplex encephalitis

Wernicke-Korsakoff syndrome Type 1 version *(64 words in Dutch)*

1. male, 67 years, admitted at the Emergency Department
2. has been found in his caravan, confused
3. he complaints about double vision
4. filthy and tremorous
5. optomotor system: restricted abduction bilaterally
6. there is a nystagmus in all positions
7. his speech is slurred
8. he cannot walk unaided
9. he is bad-tempered and obstructive
10. deep tendon reflexes are absent in his legs

Wernicke-Korsakoff syndrome Type 2 version *(64 words in Dutch)*

1. male, 67 years, admitted to the Emergency Department
2. was found in his caravan, confused
3. he complaints about double vision
4. he has a wound on his head
5. optomotor system: restricted abduction bilaterally
6. there is a nystagmus in all positions
7. his speech is slurred
8. he cannot walk unaided
9. he is bad-tempered and obstructive
10. deep tendon reflexes are absent in his legs

All items are identical, except that in the Type 2 version the patient had a head wound suggesting a traumatic disorder – which cannot, however, explain all findings. This item replaces a supportive item for the diagnosis in the Type 1 version.

List of case diagnoses:

0. Transient global amnesia *
1. Amyotrophic lateral sclerosis **
2. Giant cell arteritis
3. Carpal tunnel syndrome
4. Herpes simplex encephalitis
5. Friedreich's ataxia
6. Frontotemporal dementia **
7. Subacute combined degeneration of spinal cord
8. Cluster headache
9. Inclusion body myositis
10. Intoxication medication
11. Juvenile myoclonic epilepsy
12. Pediatric head injury with transient deterioration **
13. Lumbar disc herniation
14. Migraine
15. Multiple sclerosis
16. Myasthenia gravis **
17. Transverse myelitis
18. Neuralgic amyotrophy
19. Neuroborreliosis
20. Cervical stenotic myelopathy **
21. Progressive supranuclear palsy
22. Adie's syndrome
23. Guillain Barré syndrome
24. Brain stem stroke
25. Wernicke-Korsakoff syndrome
26. Huntington's disease

**   Practice case – not further analyzed.*

***   Pilot study only*
